# Avoidable severe morbidity from wound dehiscence after cesarean section: Practice and experience from a tertiary referral hospital in a low-income setting, Tanzania—a mixed-methods study

**DOI:** 10.3389/fsurg.2025.1524507

**Published:** 2025-09-22

**Authors:** Andrew Hans Mgaya, Raymond Oyugi Samuel, Isaya Erasto Mhando, Hery Omary Kimwela, Hans Nathanael Mgaya

**Affiliations:** ^1^ Department of Obstetrics and Gynaecology, Muhimbili National Hospital, Dar es Salaam, Tanzania; ^2^ Department of Women’s and Children’s Health, International Maternal and Child Health, Uppsala University, Uppsala, Sweden; ^3^ Department of Anaesthesiology, Muhimbili National Hospital, Dar es Salaam, Tanzania; ^4^ Department of Obstetrics and Gynaecology, St. Joseph University College of Health and Allied Sciences, Dar es Salaam, Tanzania; ^5^ Department of Obstetrics and Gynaecology, Muhimbili University of Health and Allied Sciences, Dar es Salaam, Tanzania

**Keywords:** cesarean section, wound dehiscence, surgical skills transfer, perinatal experiences, developing countries

## Abstract

**Introduction:**

This study aims to determine care-related risk factors and explore the perspectives of women and care providers about complete wound dehiscence after cesarean section at a tertiary referral and university hospital.

**Methods:**

A mixed-methods study was conducted at Muhimbili National Hospital in Dar es Salaam between April 2019 and December 2020. A case control survey compared the characteristics of interest of 131 cases of complete wound dehiscence with 393 randomly selected controls comprising cesarean deliveries between January 2015 and December 2020. In addition, six semistructured individual in-depth interviews with women, one focus group discussion with care providers, and unstructured direct observations were performed between July 2020 and December 2020. Pearson's Chi-square test and Fisher's exact test were used to determine the percentage difference of risk factors of complete wound dehiscence between cases and controls. Thereafter, a multivariate regression analysis determined the role of the independent risk factors. A thematic analysis was used to describe qualitative data.

**Results:**

Out of 524 women (131 cases and 393 controls), 75% of deliveries were performed by obstetric registrars and residents. Cases of complete wound dehiscence were more likely from cesarean deliveries performed by junior residents [odds ratio (OR) 1.8, 95% confidence interval (CI) 1.7–5.4]. Wound failure was characterized by complete wound dehiscence with intact sutures (70%) on loosely binding wound margins (62%) or avulsed from the fascial layers (38%). The perspectives of women and care providers were categorized into four themes: wound dehiscence as an indicator of the quality of care; effectiveness of clinical skill transfer and team work; maternal fear, stress, and socioeconomic burden; and significant external factors influencing care.

**Conclusion:**

Complete wound dehiscence after cesarean section was highly associated with a suboptimal surgical technique, an ineffective structure and process of clinical skill transfer, and negative experience of care from patients and their families. The identified serious and preventable gaps in the quality of cesarean section stemmed from modifiable clinical and educational practices.

## Introduction

Wound dehiscence after cesarean section (CS) remains a serious complication of mechanical wound failure (1), leading to a separation of wound margins and evisceration of bowels and other abdominal contents through the wound edges. Applying an appropriate wound closure technique remains the most important factor in preventing wound dehiscence. Regional disparities of the quality of surgical and maternal healthcare include a high incidence of wound dehiscence up to 30% in low- and middle-income countries (LMICs) (2, 3), compared with low rates (up to 4%) in high-income countries for high-risk patients (4–6). A deeper understanding of the determinants of wound dehiscence after CS is critical for improving the outcome of CS, particularly in LMICs such as Tanzania.

The risk factors of wound dehiscence after CS include perioperative infections such as chorioamnionitis and surgical site infection (7) and other perioperative illnesses such as cough and respiratory distress (8). Other contributors of post-CS wound dehiscence are maternal medical and surgical conditions that delay wound healing with or without increasing intra-abdominal pressure (9). Thus, adherence to the basic surgical training dictum of abdominal closure techniques such as suturing adequate bites of abdominal fascia (10), optimizing the suture length to wound length ratio (11), and patient-centered modified abdominal closure remain important preventive measures of wound dehiscence. Despite the advancements made in the modified abdominal closure technique, such as interrupted X-suture (12), Smead–Jones suture (13), and the application of plastic tube tension suture (14), the rate of incidence of wound dehiscence has not significantly changed over the past few decades and remains in the range of 3%–12% (2, 7, 8) and even up to 30% in some centers (3, 15) in LMICs. Therefore, because the contributing factors for wound dehiscence after CS are related to the patient, surgical techniques, and perioperative practices, an in-depth understanding of the structure and process of surgical training and skill transfer could be one of the prevention strategies for wound dehiscence in healthcare facilities (16–18).

Maternal infection is both a risk and a complication of complete wound dehiscence that highly contribute to maternal deaths in developing countries, specifically in Tanzania (19). Maternal near-miss events and the cost of care of complete wound dehiscence underscore the severity of the problem for women and the healthcare system. Multiple studies have confirmed that wound dehiscence is independently associated with perioperative factors such as anemia (20), postoperative cough, chorioamnionitis, surgical site infection (21, 22), vertical midline incision (20), and emergency abdominal surgeries (20, 22). However, some studies have refuted these patient-related risk factors (4, 7), instead implicating provider-related factors, particularly poor surgical techniques, as more significant contributors to wound failure (10–14).

In Tanzania, for the past few years since 2013, a tertiary referral and university teaching hospital recorded a three-time increase in abdominal wall repair surgeries due to wound failure after CS, of which 40%–50% were performed locally. Because high-risk pregnancies with maternal medical illness, infections, and a history of previous CS constitute the features of the main obstetric population in such referral health facilities, there was a need to perform a further analysis of care-related risk factors of wound dehiscence. In addition, assessing the determinants of surgical outcomes and experience of care in cases of wound dehiscence at a high-level referral and teaching hospital was clinically meaningful due to systematic support from medical records for performing a retrospective analysis of surgical details, wound care, infections, comorbidities, and healing outcomes. Nevertheless, the presence of multidisciplinary teams in a referral health facility ought to enable comprehensive evaluation, while the academic environment fosters a scientific approach. Findings from such settings can support the integration of recommendations into national guidelines and provide a benchmark for lower-level hospitals aiming to improve outcomes.

To our knowledge, there is limited evidence of care-related risk factors of post-CS wound dehiscence in LMICs, including Tanzania. In addition, literature on the perspectives of women and care providers about post-CS wound dehiscence is scarce. To bridge this research gap, this study aims to determine the care-related risk factors of post-CS wound dehiscence and explore the perspectives of women and care providers about complete wound dehiscence after CS at a tertiary referral and university teaching hospital in Tanzania.

## Materials and methods

### Study design and settings

A mixed-methods study was conducted at Muhimbili National Hospital (MNH) (23) during the period between April 2015 and December 2020. Using a convergent parallel design, a case–control survey conducted between January 2015 and December 2020 compared the characteristics of interest of each identified case of complete wound dehiscence after the most recent CS with three controls constituting women who were delivered by CS either 24 h before or after the cesarean delivery of a case. We also conducted six semistructured individual in-depth interviews (IDIs) with women who experienced complete wound dehiscence, between 1 July 2020 and 31 December 2020. In addition, we conducted one focus group discussion (FGD) with obstetric residents who routinely performed CS and we also carried out an unstructured direct participant observation of the structure and process of care of obstetric surgical patients, especially women with complete wound dehiscence.

MNH is a tertiary referral health facility in Dar es Salaam and a teaching university hospital serving numerous universities in Tanzania. MNH is a public hospital that offers user-fee exemptions and cost-sharing modalities for clients who are referred from public-referral hospitals. Self-referrals and referrals from private health facilities are received as private clients (either health-insured or paying services in cash) under Intramural Private Practice Management (IPPM). Between 2015 and 2020, approximately 8,200–10,500 deliveries were conducted per year for both public (60%) and private (40%) clients at a CS rate ranging from 469 to 547 deliveries per 1,000 live births.

Obstetric and newborn care took place in three maternity buildings. The first maternity building was mostly for antenatal and postnatal inpatients under the cost-sharing and user fee–exemption category. The second maternity building accommodated Reproductive and Child Health (RCH) clinic and inpatient wards for antenatal and postnatal mothers under IPPM and nursing mothers performing Kangaroo Mother Care. The third maternity building—new maternity building—served postoperative private inpatients under IPPM. The obstetrics and gynecology operating rooms were in close proximity with the maternity buildings. The operating theater building comprised of four operating rooms capable of accommodating major and minor surgeries under local, regional, and general anesthesia. Routinely two out of four operating rooms were designated for obstetric procedures that were mostly CSs. There were two working shifts for nurses and other clinical support staff. Doctors had a 24-h call duty for obstetric emergencies managed by two obstetricians (a consultant and a specialist) and two obstetric registrars or residents. The consultant on call usually stays at home during on-call duties, while the rest of the team remains in the hospital. All emergency CSs were mostly performed by specialists and obstetric registrars or residents. The hospital policy demanded all decisions for CS to be made in consultation with obstetricians. Preoperatively administered antibiotics included intravenous Ceftriaxone and Metronidazole. Generally, obstetric surgeons preferred subumbilical midline incision for emergency CSs and “Pfannenstiel incision” for elective CSs. Spinal anesthesia was preferable compared with general anesthesia, unless there was a contraindication of the former. Routinely, the abdominal rectus fascia was closed by the continuous suturing technique using “Polyglactin 910 suture” or “Polypropylene suture”—number 1. Postoperatively, women who delivered by CS were discharged on the second or third day, except when there were maternal complications or when the newborn fell sick. Wound inspection was usually performed once before the patients were discharged.

### Study population, sampling, and data collection

#### Quantitative methods

All women who had undergone abdominal wall repair because of complete wound dehiscence after the most recent CS were identified from the surgical registry in the operating room and listed. Case notes of the listed women were retrieved from the medical record storage facility. The inclusion criteria of cases were the presence of doctor notes, preoperative checklists, and operation notes during CS and abdominal wound repair after the most recent CS confirmed a separation of wound margins from the skin to the rectus fascia. Women who missed information of any inclusion criteria and those who delivered by CS outside MNH were identified using case notes and excluded. Eligible cases were recruited. The date and time of the previous cesarean delivery of recruited cases was used to identify three potential controls constituting women who delivered by CS either 24 h before or after the most recent CS of cases but without a documented history of wound dehiscence. Controls were selected within a 24-h window before or after the most recent CS of cases to reduce time-related confounding; however, there was no matching by demographic or clinical characteristics. Care-related determinants were assessed in a natural environment of routine practice. Potential controls were identified from the surgery registry in the operating theater and listed. The clinical notes of the selected potential controls were retrieved from the medical record storage facility. Thereafter, inclusion criteria similar to the one used for cases were applied. Three out of 5–21 eligible controls were randomly selected using computer-generated random numbers. The recruitment process continued until the required sample size was achieved.

The sample size was calculated using Epi info 7™ StatCalc for performing an unmatched case–control study. Assuming 50% of exposure among the control group because the true prevalence was unknown, the minimum detectable odds ratio (OR) was 2. The power of the study (1-β) was 80% at a confidence interval (CI) of 95%, and the ratio between case and control was 1–3; the minimum required sample size was 100 cases and 300 controls. We exceeded this target by including 131 cases and 393 controls to improve power and reduce potential bias.

Data were collected from 131 cases and 393 controls primarily from case notes and thereafter supplemented by the surgical registry, ward round registers, and the obstetric and neonatal database when case notes were incomplete. Thus, no eligible case or control was excluded during the study period. Information of interest included details of the most recent CS such as the date and cutting time (work hours/off hours), the level of emergency (emergency/elective), the professional level of the surgeon, and the number of cesarean deliveries performed by each surgeon before performing the CS of the studied group. The professional level of the surgeons was confirmed from the records of the office of the Head of Obstetrics and Gynaecology Department. Additional information was collected from cases, including (a) date and time of diagnosis of complete wound dehiscence; (b) intraoperative findings during abdominal wall repair including the type of suture used for closing the rectus fascia during the most recent CS, the state of sutures on the rectus fascia, and the presence or absence of SSI; and (c) maternal outcome after abdominal wall repair including blood transfusion, peripartum hysterectomy, admission to the intensive care unit (ICU), and maternal death.

#### Qualitative methods

Six semistructured IDIs were conducted with women who sustained complete wound dehiscence after CS at MNH, from July 2020 to December 2020. Purposive sampling was used to recruit women who had recovered from abdominal wound repair to explore their experiences of wound dehiscence. During recruitment, variation in age and admission category (public or private) was observed. The admission category was also generally considered a proxy for both level of care and socioeconomic status. During the month of December 2020, care providers were interviewed in FGD that comprised of seven obstetric residents. The FGD comprised of junior and senior obstetric residents and all genders with a clinical work experience of 2–5 years in general practice or within obstetrics and gynecology care.

Women were approached during hospital stay after the abdominal wall repair procedure. A verbal consent was obtained for IDIs starting from the fourth week after abdominal wall repair. Women who accepted to be interviewed were asked to provide a personal phone number and fix an appointment for the interview during postnatal clinic visits at MNH. The interview date was specifically scheduled to coincide with the postnatal care appointment date. Women were listed for interview and were asked to confirm attendance by a phone call 1 week before the interview. Six IDIs were performed by the first author. During the interviews, the companion was asked to stay outside the interview room (with the baby, when relevant). All interviews were performed in the Kiswahili language and audio-recorded and transcribed verbatim on the same day. All researchers were Kiswahili speakers (as the first language) especially when communicating with patients. However, researchers also used English when communicating among themselves and during teaching sessions, clinical meetings, and ward rounds. Interview transcripts were translated from Kiswahili to English for analysis and reporting. To ensure accuracy and contextual meaning, back-translation and validation by bilingual researchers was considered but not performed because of resource constraints.

Open-ended questions were followed up by probing on different perspectives as follows: (a) Please tell me about your experience when you saw or when you were informed of wound gapping after CS? (Probe: Thoughts? Reaction? Circumstances?) (b) What do you think was the cause of wound gapping? (Probe: Why do you think so?) and (c) What were your experiences during and after abdominal wall repair and subsequent care? (State of mind? Hospital and family duties? Social and economic circumstances?). During questioning, new perspectives that aligned with the research questions were introduced for further probing in subsequent interviews. Five interviews were adequate to reach saturation, a point at which no new thought and experience was generated. Therefore, we conducted the last interview mainly to verify information obtained from the earlier interviews.

Care providers—Obstetric residents were approached by a research assistant, a postgraduate student in public health, who completed 3 months of elective studies at MNH. The FGD composed of obstetric residents only, in order to allow openness and minimize the possibility of some individuals dominating the discussions. Participants of FGD were recruited in consideration of varying age, gender, and working experience. At the beginning of FGDs, the participants were shown a figure that presented trends of cases of complete wound dehiscence, overtime. Subsequently, they were asked to reflect and give their opinion. FGDs were conducted in a closed-door session for about 1 h. The research assistant was a passive member of the group. All FGDs were audio-recorded and transcribed verbatim. The participants used both Kiswahili and English which were the main languages employed during clinical activities.

The questions posed during the FGDs were open-ended and focused on the following topics: (a) Can you give your opinion on the trend of cases of complete wound dehiscence? (Probes: Do you have an understanding of the main causes? Your experiences when performing CS? Your views about the kind of environment that you encountered during routine work and teaching?) (b) What do you think were the main problems that women who suffered complete wound dehiscence faced? (Probes: Patents’ risk? Women's concerns? Other effects?) and (c) What could be done to reduce post-CS wound dehiscence?). Naturalistic inquiry guided the emergent analysis (24) from the initial data collection process, where FGD transcripts were read and used to identify areas that needed a verification of IDI findings and direct observation. Thematic saturation was observed when interview responses became repetitive and no new ideas or insights were identified (25). The participants were offered soft drinks during the discussions as a gesture of appreciation for participating.

It was difficult to perform adequately structured interviews with obstetricians and other care providers because of their demanding work schedule and the potential Hawthorne effect (26) from previous evidence of the effects of fear of blame and transparency associated with morbidity, which was, in turn, associated with CS (27). Therefore, we performed unstructured overt participant observations during their regular work activities, particularly during inpatient care. Such observations were also performed for inpatient surgical care, including on-call handover meetings, weekly maternal death case review meetings, teaching and service ward rounds, and obstetric operations. A notebook was used to document the structure and process of care. Perceptions and attitudes of care providers, particularly of obstetricians, were captured through informal conservations and interviews. These conversations and interviews were conducted openly and, when appropriate, recorded using an audio recorder. They also took place following participant observations of maternal near-miss or death case reviews, as well as reports of new admissions that included post-CS burst abdomen.

### Definition of terms

The cutting time of the most recent CS was categorized as “during work hours” (0800–1700h) and “off hours” (1701–0759h). For this study, the professional levels of obstetric surgeons were operationally categorized as registrars, junior residents, senior residents, junior obstetricians, and senior obstetricians. During the study, all registrars possessed a basic medical degree and a work experience in obstetrics and gynecology of less than 5 years, without postgraduate training. All residents possessed a basic medical degree and at least 3 years of clinical work experience in a surgical discipline before postgraduate training. Specifically, junior residents were in their first or second year of postgraduate training, while senior residents were in their third and final year of postgraduate training. Junior obstetricians were medical specialists who completed postgraduate training and later acquired a clinical work experience of not more than 10 years, while senior obstetricians were obstetricians with clinical work experience for more than 10 years. SSI was defined when documented in case notes as “wound infection,” “wound sepsis,” “wound discharge,” “subcutaneous abscess,” “abdominal infection/sepsis,” “postoperative sepsis/infection,” “puerperal sepsis,” or “abdominal/pelvic infection.”

### Data analysis

For quantitative analysis, data were entered and analyzed using SPSS ver. 23 (IBM, Chicago, IL). Information that was incomplete was amended after cross-checking with patient records from the surgical registry, ward round registers, and the obstetric and neonatal database. No case or control was excluded for analysis. We analyzed the percentage difference of cases compared with controls according to the professional levels of obstetric surgeons, cutting the time of the most recent CS and the number of cesarean deliveries performed by individual surgeons before performing the CS of case or control. Pearson's Chi-square test and Fisher's exact test were used to detect significant differences, when appropriate. The level of significance (α) was *p* < 0.05. Linear regression was used to assess collinearity between all independent variables predicting wound failure, in this case, obstetric surgeons' professional levels and number of cesarean deliveries performed by individual surgeons before performing the CS of the studied group. The variables were entered for performing a multivariate regression analysis with variance inflation factor (VIF) values below 5, which was indicative of no significant multicollinearity. All variables that showed significant differences between cases and controls in the bivariate analysis were entered simultaneously into the multivariate logistic regression model for further analysis to determine their independent associations with wound failure. ORs with 95% CIs were used to estimate the strength of these associations. The overall model ability of ensuring predictors significantly distinguishing between cases and controls (Omnibus Test of Model Coefficients: *p* < 0.001) and demonstrating a good fit to the data (Hosmer–Lemeshow goodness-of-fit test: *p* = 0.20) was confirmed.

Simple descriptive statistical analyses were used to present the percentage distribution of characteristics of wound dehiscence and outcomes of cases only.

For qualitative data, transcripts of IDIs, FGDs, and interview notes and recordings from unstructured overt participant observations were translated in English prior to analysis to enable report-writing and dissemination for non-Kiswahili-speaking audience and readers. After multiple readings of the transcripts, repeated similarities, patterns, and differences across the respondents were identified in a stepwise manner, and initial open coding created meaning units. These initial codes were then reviewed and grouped into broader thematic codes using the constant comparative method. Finally, we combined thematic codes into independent themes using thematic analysis (28). The notes of unstructured overt observations were summarized, categorized, and tabulated to describe aspects of clinical “handover” meetings; perioperative care for emergency and elective CS; major ward round and service ward rounds; maternal near-miss and mortality case reviews; and standards of pre- and postoperative guidelines, inpatient care programs, and hospital policy. The researchers reviewed the dataset, and the themes were reassessed for confirming their meanings and then distinguished from one another. The findings were discussed on the basis of the framework of quality of obstetric care (29) addressing the structure and process of care when CS was performed and women's experience of care during and after developing complete wound dehiscence.

### Ethical consideration

Ethical approval was obtained from the MNH Research Ethics Review Board (MNH/IRB/1/2016/21). All methods were performed in accordance with the relevant guidelines and regulations under MNH research policy. Quantitative data were collected from case notes only and the hospital policy stipulated that patients’ involvement in teaching and research activities are consented when obtaining consent for admission and treatment, considering that MNH is a university teaching hospital. Verbal consent was obtained from all participants of IDIs, FGD, and informal interviews during participant observations. Participants were informed of their right to withdraw from the study at any point and this information was kept confidential. All recordings and transcripts were anonymized before discussions within the research group and access to participants' information was given to researchers only.

## Results

### Participant characteristics and predictors of wound dehiscence

Three-quarters of the studied group (*n* = 524) were delivered by obstetric registrars or residents in the previous CS (Table 1). Junior residents delivered a higher proportion of cases (33%) compared with controls (15%) (*p* < 0.001). There was a trend of an increasing percentage of cases as the number of CSs performed by individual obstetric surgeons increased during on-call duty (*p* < 0.001). Although a majority of participants were delivered by emergency CS (86%) during the weekend and off-hours (62%), the percentage of cases was comparable to that of controls in relation to the time when CS was performed and the level of urgency. A multiple regression analysis revealed a higher likelihood of complete wound dehiscence when CS was performed by junior residents (OR 1.8, 95% CI 1.7–5.4) (Table 2). In addition, there was three times higher likelihood of wound dehiscence when obstetric surgeons had already performed seven or more CSs previously (OR 3.2, 95% CI 1.7–6.0).

**Table 1:** Distribution and percentage differences in perioperative factors during prior cesarean section by case–control status.

Factors	Total		Cases		Controls		*p*-Value
	*n* = 524	%	*n* = 131	%	*n* = 393	%	
Professional level of the surgeon							<0.001
Registrar	106	20.3	24	18.3	82	20.9	
Junior resident (Yr.1 and Yr.2)	100	19.1	43	32.8	57	14.5	
Senior resident (Yr.3)	39	7.5	13	9.9	26	6.6	
Junior specialist	100	19.1	17	13	83	21.2	
Senior specialist	178	4	34	26	144	36.8	
Level of urgency							
Emergency	443	84.5	110	84	333	84.4	0.83
Elective	81	15.5	21	16	60	15.3	
Time of CS							
Work hours	202	38.5	42	32.1	160	40.7	0.08
Off hours	322	61.5	89	67.9	233	59.3	
Number of CSs performed by the obstetric surgeon^a^							
≥7	134	25.6	54	41.2	80	20.4	<0.001
5–6	135	25.8	37	28.2	98	25.0	
3–4	128	24.4	20	15.3	108	27.4	
≤2	127	24.2	20	15.3	107	27.2	

CS, cesarean section.

^a^ CSs performed prior to the CSs of the cases or controls.

**Table 2:** Bivariate and multivariate logistic regression analyses of the likelihood of complete wound dehiscence by professional level and the number of prior CSs performed by a surgeon.

Risk factor	Case	Control	Unadjusted	Adjusted
	*n* = 131 (%)	*n* = 393 (%)	OR (95% CI)	OR (95% CI)
Professional level of the obstetric surgeon				
Registrar	24 (18.3)	82 (20.9)	1.1 (0.6–2.1)	1.3 (0.8–2.5)
Junior resident	43 (32.8)	57 (14.5)	3.2 (1.8–5.7)	1.8 (1.7–5.4)
Senior resident	13 (9.9)	26 (6.6)	0.4 (0.2–1.0)	1.9 (0.8–4.0)
Obstetrician	17 (13)	83 (21.2)	1.1 (0.6–2.1)	1.0 (0.5–2.0)
Senior obstetrician	34 (26)	144 (36.8)	1	
Number of CSs performed by the obstetric surgeon^a^				
≥7	54 (41.5)	80 (20.4)	3.7 (2.9–6.9)	3.2 (1.7–6.0)
5–6	37 (28.5)	98 (25)	2.1 (1.1–3.9)	1.9 (1.0–3.7)
3–4	20 (15.4)	108 (27.6)	1.0 (0.5–2.0)	0.9 (0.5–1.0)
≤2	19 (14.6)	106 (27)	1	

OR, odds ratio; CI, 95% confidence interval; CS, cesarean section.

^a^ CSs performed prior to the CSs of the cases or controls.

The suture material used for closing the abdominal fascial layer during prior CS of cases was mainly *polyglactin* 910 (37%) and *polypropylene* sutures (34.3%) (Table 3A). Most cases were characterized by a completely gaped wound with intact sutures (70%) that were loosely binding the wound margins (62%) or were torn from the fascial layers (38%). Seventy-five per cent of the cases presented a completely gaped wound on the 7th or later day after CS (Table 3B). Maternal near-miss events associated with complete wound dehiscence included blood transfusion (50%), peripartum hysterectomy (23%), and ICU admission (12%). Nine out of 131 patients died. The documented reasons for maternal deaths were septicemia, severe anemia, pre-eclampsia/HELLP syndrome, and peripartum cardiomyopathy. The average length of hospital stay for surviving patients was 8 days (ranging from 2 to 78 days).

**Table 3A:** Percentage of intraoperative characteristics of wound dehiscence cases.

Intraoperative characteristics	Number (*n*)	Percentage (%)
What suture material was used?		
Polyglactin 910 suture	49	37.4
Polypropylene suture	45	34.3
Braided-coated polyglycolic acid suture	4	3
Silk	2	1.5
Missing record	13	9.9
What was the state of the suture?		
Broken suture	24	18.3
Unbroken suture	92	70.2
Missing record	15	11.5
What was state of unbroken sutures on apposed margins?		
Loosely binding wound margins	57	62.0
Avulsed from fascial margins	35	38.0
Was there surgical site infection?		
Yes	35	26.7
No	69	52.6
Missing record	26	19.8

**Table 3B:** Percentage of postcesarean section outcomes of wound dehiscence cases.

Postcesarean section outcomes	Number (*n*)	Percentage (%)
When did wound gapping occur, postoperatively		
Earlier than day 3	5	3.8
Day 3–4	12	9.2
Day 5–6	16	12.2
Day 7 or later	98	74.8
Was there a history of blood transfusion?		
Yes	66	50.4
No	65	49.4
Was hysterectomy done?		
Yes	30	22.9
No	101	77.1
Admission to ICU		
Yes	20	15.3
No	111	84.7
Maternal deaths		
Yes	9	6.9
No	122	93.1

We identified four themes that we deemed to influence or reflected the perception and attitude of women and care providers toward the experience of care of postcesarean wound dehiscence: (1) wound dehiscence as an indicator of the quality of care, (2) effectiveness of clinical skill transfer and team work, (3) implicit maternal fear, stress, and socioeconomic burden, and (4) significant external factors influencing care.

### Wound dehiscence as an indicator of the quality of care

We observed adherence to accessible perioperative guidelines and checklists and the use of infection prevention and control (IPC) tools during routine care of post-CS wound dehiscence. Although the rate of complete wound dehiscence was documented and considered a key performance indicator (KPI) of the quality of care improvement (QI), neither were the surgical KPIs accessible nor routinely analyzed and feedback provided to all care providers. Thus, care providers did not seem to recognize wound dehiscence as a measure of quality of surgical care. During an on-call handover meeting, one stated:

But I remember discussing this, one time, and we found the occurrence of burst abdomen at less than 1%, so it may be very low. Why discuss? (Senior Obstetrician)

Although care providers had a divergent opinion regarding the trend of post-CS complete wound dehiscence (Supplementary Table S1), both women and care providers negatively perceived the risks associated with the complications of post-CS wound dehiscence:

I was very afraid of dying. (Woman 35-yr private patient, IDI 1)

These mothers suffer a lot especially the young ones who lose their uterus. (Junior Resident 7, FGD)

There was a belief that the structure of the health system and the changing features of the obstetric population highly contribute to high rate and risk of wound dehiscence at the highest referral health facility-MNH. Furthermore, some care providers perceived an unavoidable risk of wound dehiscence during CS of women with multiple uterine scars:

Some (women in labor) come here with three previous scars; you cannot clearly distinguish the rectus from the scar (Senior Resident, FDG).

Postoperatively, care providers were observed to be supportive to women with wound dehiscence (Supplementary Table S2). All women interviewed were gratified by the guidance, encouragement, and support received from the care providers. However, their psychological condition was not routinely reported postoperatively, except in cases of puerperal psychosis. Thus, care providers perceived wound dehiscence among the common surgical complications within the whole health system but considered it less concerning at MNH compared with low-referral facilities:

You can find that cases of burst abdomen after CS from referral hospitals are more than those from our hospital (MNH). (Junior Resident 3, FGD)

### Effectiveness of clinical skill transfers and team work

We observed a uniform practice of the abdominal closure technique for all CSs that were rarely modified on a case-to-case basis based on patients’ risk of wound failure (Supplementary Table S2). During clinical meetings, most of the emergency CSs were reported to have been performed by one or two obstetric residents (i.e., 4–16 CSs in a 24 h on-call duty). Care providers were confident of their ability to perform safe CSs but expressed concerns that surgical trainees, particularly residents, were not adequately skilled in abdominal wall closure. There was also a divergence of opinion on whether heavy workload and severe physical exhaustion during on-call duties compromise surgical performance or provided an opportunity to gain surgical experience:

Some perform these operations (Caesarean sections) when they are too exhausted and lack concentration. (Senior Resident 4, FGD)

Managing many cases is good for acquiring skills. (Junior 0bstetrician)

Despite a hierarchal consultation procedure from junior to senior doctors on call outlined in a widely accessible on-call duty roster, we observed obstetric residents preferentially seeking assistance from one another rather than from specialists when encountering difficulties during surgery. However, there were divergent opinions regarding the working relationship of care providers; some expressed reluctance to engage with their seniors out of fear of being labeled incompetent; others vented their frustration on the limited availability of specialists during routine clinical activities:

Juniors are busy, and seniors are also busy. There is not enough time for skill transfer (Junior Obstetrician)

Although most of the clinical activities were specialist-led, an evaluation of surgical activities of residents was commonly performed retrospectively using a log book. During maternal near-miss and death case reviews, care providers commonly flagged lost opportunities for practical skill transfer and assessment of junior doctors, as well as limited adherence to standard guidelines.

For a clinician, the time to teach is when you are doing the ward rounds or in theater, but here (MNH), students learn from discussing cases of maternal complication or death. (Senior Obstetrician)

### Implicit maternal fear, distress, and socioeconomic burden

Women's unmet expectations during care seem to have caused traumatic experiences in them, provoking feelings of sadness and worry. We witnessed occasions where some were distressed by the experience of perioperative pain after abdominal wall repair, and others resisted undergoing reoperation for abdominal repair (Tables 3A,B). All women expressed a kind of fearful distress over the risk of death, when informed of wound failure. Wound pain, evisceration, and hysterectomy were the worst distressful events:

Operations are dangerous. Everyone (relatives at home) was stressed (Woman 35-yr private patient)

Despite a nurse-led counseling for distressed patients, a pain assessment scale and a protocol for breaking “bad news” were not instituted. However, care providers expressed concern for life-threatening maternal clinical complications such as wound infection, septic uterus, anemia, and risks associated with prolonged immobilization. Care providers’ discussions primarily focused on maternal complications (including burst abdomen) and risk of deaths during maternal near-miss and death case review sessions. However, the psychosocial and economic burden faced by women was rarely discussed and, in fact, was overlooked. As noted by some:

They (women) get all sorts of problems especially from wound sepsis, AKI (acute kidney injury), anemia, and other problems from immobility of postoperative patients. (Junior Obstetrician)

Obviously, they have poor wound healing from anemia and poor ambulation, also may get uremia from AKI (Junior Resident 3, FGD)

We observed that most of the postoperative patients who stayed for more than 72 h without any maternal complications had either a sick newborn in the neonatal ward or were detained because of unpaid hospital bills. Both women and care providers expressed concerns of the cost burden of hospitalization, reoperation, and support from family members:

Staying meant more money to pay. I could not afford. (Woman 32-yr-private patient)

Sometimes they have no social support, even the partner run away… (Junior Resident 7, FGD)

There was a clearly laid out hospital social welfare policy to address the postoperative problems of patients, including a lack of social and financial support, with the provisions of service cost exception, when appropriate. On average, we observed that patients with wound failure had a prolonged hospital stay of at least 10 days. Some post-CS and abdominal repair patients perceived hospital stays as both a financial burden and a risk of loss of earnings:

He (Husband) came too but not a lot. He had to work. … my small business had to be closed. (Woman 36-yr public patient, 3)

### Reputed external factors influencing care

During case reviews and ward rounds (Supplementary Tables S1, S2), inquiries into the possible reasons for wound failure was largely limited to patient-related factors: obesity, post-CS SSI, perioperative illness (anemia, cough, vomiting, ascites, uremia, etc.), presence of multiple abdominal scars, post-CS immobility, and expired sutures. However, both women and care providers agreed that care providers play a role in preventing wound failure:

Any surgeon can face complications. The problem is if you took precautions or not (Senior Resident 3, FGD)

We found a clear and accessible structured 24-h duty roster for doctors managing obstetric and gynecological emergencies consisting of two specialists, three residents or registrars and intern doctors, supported by nursing and other support staff. Even so, women perceived that the choice and safety of their care was beyond their control, especially in emergency situations:

When you come in at night you do not have a choice, we meet any Doctor (Woman 41-yr public patient, 5)

On the other hand, care providers perceived the risks of wound dehiscence as primarily relating to individual patient characteristics, and therefore, they were beyond their control:

There are many reasons for burst abdomen after CS. It may be coughing, vomiting, excessive abdominal fat and others. (Junior Obstetrician)

Although clinical supervision and mentoring of low-referral facilities was seldom performed, there was an established clinical supervision and mentoring program for MNH in lower referral facilities. Care providers appeared to believe that most cases of wound failure were referrals from other facilities, but this came without any supporting statistical evidence. Both women and care providers regarded such cases as largely beyond their control:

I had a lot of fat. (…) I also coughed a lot. (Woman 35-yr private patient, 1)

This is the highest level of referral system. This could be expected for complicated cases… CS (Senior Obstetrician)

We think that when women felt vulnerable after experiencing complications of wound dehiscence (such as evisceration and hysterectomy), they adapted to and accepted the situation by invoking religious faith and placed their hopes on divine healing:

I was praying not to go back for another operation (…) I did not think I would have survived. (Woman 41-yr public patient)

## Discussion

### Main findings

This study found a high risk of complete wound dehiscence when CSs were performed by junior obstetric surgeons within the structure of care at a university teaching hospital. The risk of wound dehiscence increased when surgeons, in this case mainly residents, endured physical exhaustion after performing as many as seven or more CSs during on-call duty. This aligned with the intraoperative findings during abdominal wall repair that suggested poor abdominal closure technique as the main reason for wound failure. On the other hand, limited surgical competency, coupled with structural and process-of-care deficiencies (30), could contribute to wound failure. Furthermore, the perceptions of women and care providers underlined how the structure and process of clinical care led to inadequate clinical teaching, surgical skill transfer and team work, and a negative patient experience of care. Our findings highlighted an opportunity to enhance care providers' involvement in standardizing care based on KPI and reorganizing the process of skill transfer from senior to junior doctors.

### Standards of structure and process of clinical teaching and care

During the study period, it was found that prior clinical experience and surgical skills of most junior residents were acquired from working in primary or low-referral health facilities with limited supervision and mentoring due to understaffing, poor communication, lack of participation in decision-making, and limited understanding of mentoring and supervisory roles (31, 32). It is probable that obstetric registrars and senior residents might have better post-CS outcomes than their junior counterparts because of working at the study site for at least 2 years. Although complete wound dehiscence was one of the KPIs for maternal healthcare quality, limited access to hospital KPI and standard operating procedure (SOP) documents, along with evidently minimal performance evaluation and feedback (32, 33), may have compromised its recognition as a quality indicator. Subsequently, not recognizing wound dehiscence as a quality indicator might have led to an underemphasis of applying the appropriate abdominal closure technique.

Perioperative care appeared standardized and structured with a high surgical output; however, the working relationship between senior and junior doctors suggested potential dysfunction, including poor communication and limited participation in decision-making processes—issues also reported previously within the study setting (26, 32) and in similar settings elsewhere (34, 35). Thus, there was a lost opportunity for objectively assessing the surgical skills of junior doctors and subsequently imparting demand-driven surgical training. We cautiously used the professional level of surgeons as a proxy for surgical experience in abdomen wall closure, with the knowledge that a measure of surgical experience should also include the ability to work as a team, leadership skills, stress control, clinical knowledge, and attitude, with a discussion of these factors being beyond the scope of this study.

A high risk of post-CS wound failure was associated with seven or more individually performed CSs regardless of whether the previous cesarean delivery took place during working hours or off hours. Unlike other studies (36, 37), we found divergent opinions on whether a high surgical workload for an individual surgeon ultimately improved their surgical skills. Despite the desire of obstetricians to increase the surgical workload for residents to maximize surgical exposure, a contextual analysis is required to assess the potential inhibitive effects of inadequate team work and leadership within the teaching environment. Given the benefits of systematically regulating duty-hour restrictions (38), interventions to improve surgical teaching and skill transfer should positively impact the work experience of care providers and subsequently improve patient care.

We found junior doctors often managing complicated surgical cases independently, with reluctance to consult their seniors. This lack of supervision not only compromised their adherence to institutional standards of practice but also limited the opportunities for making an objective assessment of their surgical performance. Aligning with previous findings (39), a lack of adherence to a structured teaching agreement between academic and clinical staff within the framework of SOP of clinical care might have imposed a risk of substandard surgical teaching and practice. Based on our study finding regarding the characteristics of dehiscent wounds, a poor surgical suturing technique was likely the main contributing factor, as perceived by both women and care providers. In tune with other consistent evidence from previous studies (30, 32, 39), there was a demand for improving surgical skill training and clinical supervision of junior staff. This may include formalizing a chief residency period, an intermediary period bridging the clinical gap between junior doctors and specialists, and enhancing hands-on skills transfer, mentorship, and leadership (40, 41), while fostering peer learning, psychological safety, and open communication when encountering difficult clinical decisions and adverse clinical events.

### Women and care providers’ experience of care

Post-CS wound dehiscence was associated with near-miss events such as blood transfusion, peripartum hysterectomy, and admission for intensive care that contributed to maternal fear and distress from pain and subsequent prolonged hospital stay. Care providers' awareness of these negative effects could have caused the fear of blame and defensive reactivity (27), with the claim that wound failure was part of the learning process and therefore was beyond their control. On the other hand, a lack of structured pain assessment and psychological care might have imposed a risk of women succumbing to severe psychological distress and hence resorting to adaptive self-blame (42) by implicating their perioperative health status (such as cough, vomiting, obesity, etc.) as the cause of wound failure. Similarly, without an effective means to objectively measure and intervene in pain and psychological distress, women's experience of care may go unrecognized, potentially leading to a loss of hope in recovery, reliance on religious beliefs, and divine healing. Our findings highlighted the importance of considering women's experience and perspective of care when designing interventions to improve the multidimensional aspects of maternal healthcare in a real world and natural environment of care.

Despite the existence of a well-established institutional social welfare policy for inpatients in need of social and financial support, prolonged hospitalization after abdominal wall repair continued to impose unnecessary cost burden and loss of earnings to patients and their families. Both women and care providers confirmed the endured negative psychosocial and economic consequences that might have changed their focus from hospital-based care to a request for premature discharge. Improving surgical safety and delivering patient-centered care that minimizes the risk of surgical complications are critical to preventing the negative experience of care and imposing unnecessary financial burden on women and their families, both in other regions of Tanzania (43) and broadly in LMICs (44–46).

This study aimed to assess care-related risks of wound dehiscence during and after CS in a natural practice environment, enabling the identification of practical interventions to improve provider practices and overall quality of care. Avoiding strict matching of clinical characteristics prevented potential masking and overcontrolling of care-related exposures under routine conditions. Moreover, unmeasured or unidentified factors could still have confounded the results, even if the cases and controls were exactly matched for potential confounders. On the other hand, exact matching was constrained by incomplete or inconsistent definitions of clinical characteristics in case notes and the rarity of CS-related comorbidities (e.g., anemia, chorioamnionitis, perioperative complications) (21), which could have reduced the sample size, limited the number of controls, and introduced selection bias.

### Study strength and limitations

The mixed-methods approach provided an opportunity for combining a quantitative assessment of risk of post-CS wound dehiscence and the perspectives of women and care providers, with insights from women and care providers helping to explain the identified risks and informing the design of optimal interventions. Thus, the findings highlighted the true picture of the determinants of complete wound dehiscence. The quantitative survey compared the added risk of wound dehiscence of one case vis-a-vis three controls who were randomly selected, hence increasing the power of the study and minimizing the risk of selection bias. The STROBE checklist was used to strengthen the reporting of the quantitative component, while the COREQ checklist was used to ensure the trustworthiness, relevance, and transferability of qualitative methods and findings. IDIs, FGD, and unstructured overt direct observations provided a deep understanding and corroborated women's and care providers' point of view of care received during and after CS. The interviews purposefully involved women of different age groups and service categories (public/private category) and care providers of different genders and work experience, in order to achieve diversification and facilitate a credible discussion. Kiswahili language was used for achieve conformity with the natural environment of maternal care. The research team determined what observational data should be collected and how to collect them, based on maternity care routines of interest. Performing an unstructured overt observation with an audio-reading device proved economical, offering the flexibility of data collection across times of day or weeks, with minimum observer bias. We believe that our study findings are generalizable in teaching health facilities that provide CEmOC especially in LMICs.

Despite the aforementioned strengths, our study had limitations such as interpretation of quality of care without considering the effect of observer bias due to the variable quality of documentation and subsequent supplementation of missing information. The professional levels of care providers were a proxy for surgical competence and skills that required a more complex assessment. In addition, a bigger sample size could have increased the number of CSs performed by all groups and hence might have evened-out the probability of encountering wound failure across all obstetric surgeons. Unaccounted inter- and intraoperative breaks might have reduced care providers' risk of pain and physical or mental fatigue. Therefore, the assessed risk of surgical errors based on endured surgical workload should be taken with a note of caution. In addition, in the qualitative methods, the unstructured overt observation imposed a risk of the Hawthorne effect (26) because the time of data collection was short, and there was also a risk of losing context in between the observations (47). Data were translated from Kiswahili to English without back-translation, which may have led to a loss of nuanced meanings and could affect the validity of the findings.

## Conclusion and recommendations

Complete wound dehiscence after cesarean section was highly associated with the suboptimal surgical technique, an inadequate structure and process of clinical teaching, and the negative experience of care by patients and their families. The identified care-related gaps in the quality of CS stemmed from modifiable clinical and educational practices. Improving the safety of CS requires a comprehensive approach that addresses both the technical and the experiential dimensions of care, particularly in LMICs. Therefore, we recommend the following:

a. Making a systematic assessment of clinical teaching practices that evaluate access and opportunities for clinical and surgical skill transfer in a complex clinical environment. Such assessments must determine what works and what does not when implementing policy-guided clinical teaching, including but not limited to mentor–mentee coaching, surgical supervision, and team-based learning approaches.
b. Strengthening surgical training through structured, stepwise positively re-enforcing learning models that integrate simulation-based hands-on training for junior doctors and ensuring real-time surgical supervision by senior clinicians, including residents, thus creating a learning platform that fulfills the professional needs of care providers and also ensuring patient safety.
c. Increasing objectivity in evaluating the clinical and surgical competence of health practitioners/trainees by focusing on knowledge, surgical skills, as well as adherence to standardized practice, the ability to work within a multidisciplinary team, good leadership, stress control, and overall attitude toward continuous learning.
d. Establishing a formal Chief Residency period for all senior residents to serve as a bridge between junior doctors and specialists. This role will enhance hands-on skill transfer, mentorship, leadership development, and representation of residents in departmental committees and meetings, while also fostering peer learning, psychological safety, and open communication, particularly during adverse clinical events.
e. Establishing and institutionalizing a continuous QI culture that prioritizes patient safety and the multidimensional aspects of the quality of care, including the structure, process, and experience of care. Continuous monitoring, feedback loops, and inclusive participation from all cadres of healthcare providers are essential for sustaining improvements in the quality of cesarean section care.
f. Implementing specific QI interventions with the compounding effect of preventing wound failure and the negative perioperative experience of care, including but not limited to pain management, prolonged hospital stay, and cost burden from postoperative care.

## Data Availability

The raw data supporting the conclusions of this article will be made available by the authors without undue reservation.
