# Supplementary material for: Avoidable severe morbidity from wound dehiscence after cesarean section: Practice and experience from a tertiary referral hospital in a low-income setting, Tanzania—a mixed-methods study
Source: Front Surg. 2025 Sep 22;12:1524507. doi: 10.3389/fsurg.2025.1524507 (PMC12497777; doi:10.3389/fsurg.2025.1524507)
Supplement: Supplementary file 1 [file Datasheet1.pdf]

Supplementary table 1. Summary of findings during unstructured participant observations

**'Hand over' clinical meetings**	**Perioperative care for emergency and elective CS.**	**Major ward round and services ward round**	**Maternal near miss and mortality case reviews**	**Standard pre- and post-operative guidelines, policy and programs**
Wound failure as a quality of care indicator<br><br>Initial management and progress of all admissions and deliveries in the past 24 hours was reported and discussed.<br><br>Progress of inpatients in the ICU was report and discussed.<br><br>Readmissions within 48 hours were discussed with concern of being discharge prematurely<br><br>Cases of complete wound dehiscence were reported as part of new admissions but rarely followed up as part of quality improvement (QI).	Perioperative care was guided by SOP and check lists<br><br>Adherence to preoperative and post-operative care was observed.<br><br>Surgical instrument for abdominal fascia closure was present<br><br>IPC tools were in place	Abdominal wound assessment was performed for all post CSs and post laparotomy patients<br><br>Surgical notes and post-operative orders were not standardized<br><br>Quality of surgical documentation was sometimes reported to be poor during QI evaluation<br><br>Cases of wound failure were not documented for QI evaluation as it was done for cases with bed score, wound sepsis, iatrogenic injuries etc.	A specialist led the meeting. Head of department of obstetrics and gynaecology and maternity build managers present.<br><br>The meetings were attended by majority of obstetricians, residents and registrars, nurses and medical students. QI officers and pharmacy and radiology staff, and a doctor from 1-2 referring hospitals was rarely present.<br><br>Care providers were concerned of severe morbidity associated with wound failure but regarded the event as rare and not a QI problem at MNH; *'…we found the occurrence of burst abdomen at less than 1 %, so may be very low. Why discussing?'*	Accessible hard copies of perioperative guideline and check lists<br><br>Presence of surgical KPI document with limited accessibility. Analysis of surgical outcome include wound dehiscence was not included.<br><br>Hospital surgical quality indicators were established but not easily accessible and hence unknown to some care providers, including obstetric surgeons.<br><br>Wound failure was not a notifiable surgical quality

				indicator as SSI, cancellation of surgery etc.
<u>Effectiveness of adequacy of skill transfers and team work</u><br>In a 24hours-work shift, most of emergency CSs were performed by one or two obstetric residents (i.e. 4 to 16 CSs).<br><br>Obstetricians were commonly concerned of not been consulted by obstetric residents during management of seriously ill patient or difficult or operation<br><br>Obstetric residents and obstetricians were required to clarify questionable processes of patient management e.g. reason for CS, low Apgar score, maternal death.<br><br>The meetings were mostly attended by doctors and rarely nurse, who were also handling over patients in the ward or were attending a seminar.	Abdominal closure technique was standardized for all CS, and rarely modified based of patients-risk of wound failure<br><br>Obstetric residents, preferred assistance from fellow residents compared to obstetricians during surgery.<br><br>Most obstetricians performed CS, for private patients, and rarely performed CS as a surgical demonstration to junior doctors.<br><br>Assessment of obstetric residents' surgical activity was commonly performed retrospectively by scoring and signing in a log book	Ward rounds were attended by specialists, obstetric residents and registrars, nurses, interns and medical students.<br><br>All major ward rounds were led by specialists (senior or junior obstetrician), but service ward rounds were occasionally led by senior residents.<br><br>Review noted of post CS wound dehiscence were mostly focused on patient-related risk factors and subsequent care<br><br>Surgical competence during CS mostly focused on avoiding intraoperative complications (e.g. severe bleeding, iatrogenic injuries and sepsis)	Wound failure was considered a surgical learning process; *'Getting a burst abdomen, we can say is part of a learning process'*<br><br>Occasionally, consulting a senior my led to criticism; '*When you are a resident you may be in a dilemma. Asking too much help seem to be as incompetence and some seniors may label you'*<br><br>Ethical aspects such as beneficence of care, patients' autonomy, and doctors' ability to decide on patients' behalf, lifesaving situations were discussed.<br><br>Postmortem was recommended for all cases that died within 24hours from admission or after obstetric surgery, and in case of an anticipated medical litigation	Limited surgical training programs and simulators for hands-on training<br><br>Nurses-led quality improvement activities including assessment of surgical outcomes<br><br>The established policy of specialists-led management was observed, but was more enforced by nurses than doctors.<br><br>Department policy of real time recording and assessment of clinical work performed by obstetric residents was established.

<u>Women's psychosocial and economic burden</u><br><br>Care providers advised performing hysterectomy, in cases with wound dehiscence with or without evidence of infected uterus<br><br>Patients with puerperal psychosis and a concomitant serious physical illness (e.g. sepsis, stroke,) were reported.<br><br>Psychological state and concerns of postoperative patients was not routinely reported<br><br>Postoperative patients more than 72 hours and others who were retained to clear the hospital bill was not reported	After diagnosis of wound dehiscence, women were fearful dying and complications of re-operation<br><br>Some women refused re-operation for abdominal repair, until they were convinced by other hospital staff.<br><br>Some women cried in severe pain during wound care after CS or abdominal repair.<br><br>Some post CS and post abdominal repair cases were retained after being discharged to clear hospital bill	Care providers asked patients: 'how they are doing', if medications were given/taken and 'if they have other concerns'?<br><br>Post abdominal repair patients were informed of reasons for re-operation (e.g. perioperative illness, obesity and immobility)<br><br>Most of the patients with wound failure had a prolonged hospital stay (on average 10 days)<br><br>Some post CS and post abdominal repair cases were distressed and requested assistance to clear the hospital through social welfare office	Maternal near miss review reasons highlighted maternal complications (including burst abdomen) and risks of maternal deaths<br><br>Women's psychosocial and economic burden was rarely discussed: '*They (women) get all sort of problems especially from wound sepsis, AKI (acute kidney injury), anemia, and other problems from immobility of post-operative patients'*	Pain assessment scale and protocol for breaking 'Bad news' to the patients was not available for post-operative pain management<br><br>Nurse counselors and psychologist/psychiatric were accessible for care of psychologically distressed patients<br><br>Criteria of consulting Nurse counselors and psychologist /psychiatrist was unclear. (Lack of clear protocol)<br><br>Social welfare officer enforced hospital policy for addressing post-operative patients' problem including lack of social and financial support for patients who did not visiting relatives and others who failed to pay hospital bills.

Reputed external factors influencing care<br><br>Line of inquiry of possible reasons for incidental wound failure was commonly limited to patient related factors: obesity, detected post CS SSI, perioperative illness (anaemia, cough, vomiting, ascites, uremia etc.), presence of multiple abdominal scar, post CS immobility and expired sutures.<br><br>During observations, care providers highlighted that case of wound failure were mostly admitted as referrals (without statistical evidence).	Wound failure was reported to occur to women who were at risk of poor wound healing.	Inadequate antenatal care and patient related risk factors were mainly discussed reasons for wound dehiscence (e.g. maternal infection, multiple abdominal surgical scars, obesity, cough, respiratory distress, anaemia, HELLP syndrome and immobilization for post CS under ICU care)	Wound failure was presumed unavoidable at highest referral facility with patient population with multiple previous abdominal scars, choriamnionitis, CS with huge myomas, ascites etc. '*This is the highest level of referral system. This could be expected for complicated case, not elective CS.*'	Clinical supervision and mentoring program for low-referral facility was established<br><br>Clinical supervision and mentoring visits were seldom performed
