# Supplementary material for: Avoidable severe morbidity from wound dehiscence after cesarean section: Practice and experience from a tertiary referral hospital in a low-income setting, Tanzania—a mixed-methods study
Source: Front Surg. 2025 Sep 22;12:1524507. doi: 10.3389/fsurg.2025.1524507 (PMC12497777; doi:10.3389/fsurg.2025.1524507)
Supplement: Supplementary file 2 [file Datasheet2.pdf]

Supplementary table 2. Summary of converging and diverging opinion of women and care providers

Converging opinion	*Women's' opinion*	*Care providers' opinion*
**Wound failure as a surgical quality indicator**<br>Complete wound dehiscence is associated with severe morbidities and poor experience of care.	Experience of fear and distress of uncertainty of survival	Rate of wound dehiscence was low hence disregarded as problem at MNH<br><br>Some women were reported to undergo post CS hysterectomy, a maternal near-miss event
**Effectiveness of skill transfers and team work**<br>Maternal complications were regarded as the main contributors of wound dehiscence	Women were not confident with all care providers 'skills in an emergency situation.	Care providers had a diverged opinion whether overworking by performing many CS was a risk of surgical error, or a pre-requisite for surgical competence<br><br>Lack of leadership diplomacy when teaching obstetric residents who did not want to be labelled incompetent hence seldom consulted obstetricians
**Implicit maternal psychosocial and economic burden**<br>There were concerns regarding cost of prolonged hospitalization and re-operation<br><br>Wound failure was a result of women characteristic – obesity and perioperative illness	Women expressed depended on family members and had strained relationship with partner and family members.<br><br>Women were distressed with wound failure and some resorted to self-blame.	Care providers highlighted physical complications (e.g. SSI, renal failure and immobility, adding to postoperative complications
**External factors affecting process and experience of care**<br>Both women and care provider regarded childbirth carries unpredictable complications	Women expressed loss of hope for survival and relied on 'Godly' and 'divine' healing	Encountered Post CS complete wound dehiscence was regarded as part of learning.<br><br>Care providers thought that wound failure was common among referral patients

		Wound failure was anticipated in case of maternal infection and multiple previous caesarean scars
